# Comparative Outcomes of Reinforced Tension‐Line Sutures Versus Standard Closure Techniques in Patients Undergoing Laparotomy: A Systematic Review and Meta‐Analysis

**DOI:** 10.1111/ans.70439

**Published:** 2026-01-14

**Authors:** Rama H. G. Mikhail, Shahin Hajibandeh, Shahab Hajibandeh, Marty Smith, Ee Jun Ban, Rodney Jacobs, Siobhan C. McKay

**Affiliations:** ^1^ Hepato‐Pancreatico‐Biliary and General Surgery Unit Northern Health Epping Victoria Australia; ^2^ Hepatobiliary and Pancreatic Surgery and Liver Transplant Unit Queen Elizabeth Hospital Birmingham Birmingham UK; ^3^ Department of Hepatobiliary and Pancreatic Surgery Manchester Royal Infirmary Hospital Manchester UK; ^4^ Department of HPB Surgery Alfred Health Melbourne Australia; ^5^ Institute of Cancer and Genomic Science University of Birmingham Birmingham UK

**Keywords:** incisional hernia, laparotomy, reinforced tension‐line suture

## Abstract

**Background:**

The reinforced tension‐line suture (RTLS) technique distributes mechanical stress more evenly than traditional closure when closing a laparotomy wound, potentially reducing incisional hernia (IH) risk. We aimed to compare outcomes of RTLS versus standard closure techniques in patients undergoing laparotomy.

**Methods:**

Systematic search of PubMed, MEDLINE, Web of Science, and bibliographic reference lists was conducted (last search: 26 January 2025). The protocol was registered with PROSPERO. Comparative studies reporting outcomes of RTLS versus other closure methods were included and their risk of bias was assessed. IH, Clavien‐Dindo (C‐D) ≥ III complications, wound dehiscence, wound infection, and procedure time were the evaluated outcome measures. Odds ratios (OR) for dichotomous outcomes and mean differences (MD) for continuous variables were determined. Heterogeneity was assessed using I^2^ and Cochran's *Q* test.

**Results:**

Five comparative studies (four randomised and one observational) enrolling 708 patients who had their laparotomy wound closed using RTLS (*n* = 393) or standard closure (*n* = 315) were included. Use of RTLS significantly reduced risk of IH compared to standard technique (5.6% vs. 18.1%, OR 0.24; 95% CI: 0.15–0.38; *p* = 0.005). However, no significant differences were found in C‐D ≥ III complications (10.2% vs. 3.2%, OR 0.81; 95% CI: 0.18–3.54, *p* = 0.62), wound dehiscence (2.3% vs. 6.9%, OR 0.34; 95% CI: 0.06–1.84, *p* = 0.62), wound infection (9.0% and 10.4%, OR 0.34; 95% CI: 0.06–1.84, *p* = 0.62) or procedure time (MD 23.50; 95% CI: −59.88–106.87, *p* = 0.16) between two groups.

**Conclusions:**

RTLS seems to significantly reduce IH incidence after laparotomy without increasing post‐operative morbidities or procedure time. Further Level 1 evidence is needed.

## 1 Introduction

Despite advancements in surgical techniques, post‐operative incisional hernia (IH) remains a common and significant complication of abdominal surgery occurring in up to 38% of cases [1]. Patient comorbidities, emergency surgery, previous midline incision or wound contamination are known to increase the risk of IH [1, 2]. Alongside the impact to a patient's quality of life, there are considerable healthcare costs associated with treatment of incisional hernias, with nearly 900 000 IH repairs performed in Australia alone in the last 20 years [3]. Incisional hernias can be associated with severe complications such as incarceration, necessitating emergency surgery. Various strategies have been proposed to mitigate the risk of post‐operative IH, such as the use of modified suture closure or application of prophylactic mesh in high‐risk patients [4, 5, 6, 7]. Despite these developments, there remains a gap in technique consistency and clinical adoption which, coupled with a lack of consensus on ideal patient selection criteria, has limited widespread application. Ultimately, a refined approach to wound closure is required and involves consideration of a modified technique for high‐risk patients/operations including the type of suture, the technique of closure as well as careful tissue handling.

The reinforced tension‐line suture (RTLS), first described by Hollinsky et al. [8] in 2007, is an alternative to the traditional primary suture closure of abdominal wounds that aims to evenly distribute mechanical stress, thereby reducing the risk of IH. Despite the proposed benefits, there has not been broad adoption of the technique. Despite the existence of comparative studies, no evidence synthesis evaluated comparative outcomes of RTLS and other closure techniques in patients undergoing laparotomy.

This comprehensive systematic review and meta‐analysis aims to evaluate comparative outcomes of RTLS and standard technique in patients undergoing laparotomy.

## 2 Methods

### 2.1 Registration and Protocol

The study adhered to a pre‐defined protocol registered in PROSPERO (CRD420251001905), an open international database for prospectively registered systematic reviews.

### 2.2 Study Design and Eligibility Criteria

The standards of Preferred Reporting Items for Systematic Reviews and Meta‐Analyses (PRISMA) statement [9] were respected in the methodology of this study. All existing randomised and observational studies which evaluated outcomes of RTLS compared to standard abdominal closure were considered. Single‐arm studies, expert opinions, letters to editors, or case reports or series were not considered.

### 2.3 Population

All adult patients (age more than 18) who underwent a midline laparotomy closure with RTLS compared to standard abdominal closure were included.

### 2.4 Intervention and Comparison

The use of the RTLS technique was considered the intervention of interest which was compared to the standard abdominal closure technique.

### 2.5 Outcomes

The primary outcome measure was the occurrence of a post‐operative IH during follow‐up, defined as the formation of a hernia at the surgical site after the closure of an abdominal incision. Secondary outcome measures included Clavien‐Dindo > III complications, wound dehiscence, wound infection and procedural time.

### 2.6 Literature Search Strategy

A strategy for literature search was formulated and run via PubMed, MEDLINE, Web of Science (Appendix A). The keywords ‘reinforced tension‐line suture’, ‘*incisional hernia*’, ‘*abdominal surgery*’, ‘*prevention*’ and ‘*technique*’. MeSH headings were utilised where appropriate. Boolean operators were also used. An independent evaluation of reference lists of the identified studies or reviews was carried out by two independent authors (RM and SCM). The most recent literature search was performed on January 26 2025.

### 2.7 Study Selection

**FIGURE 1:**
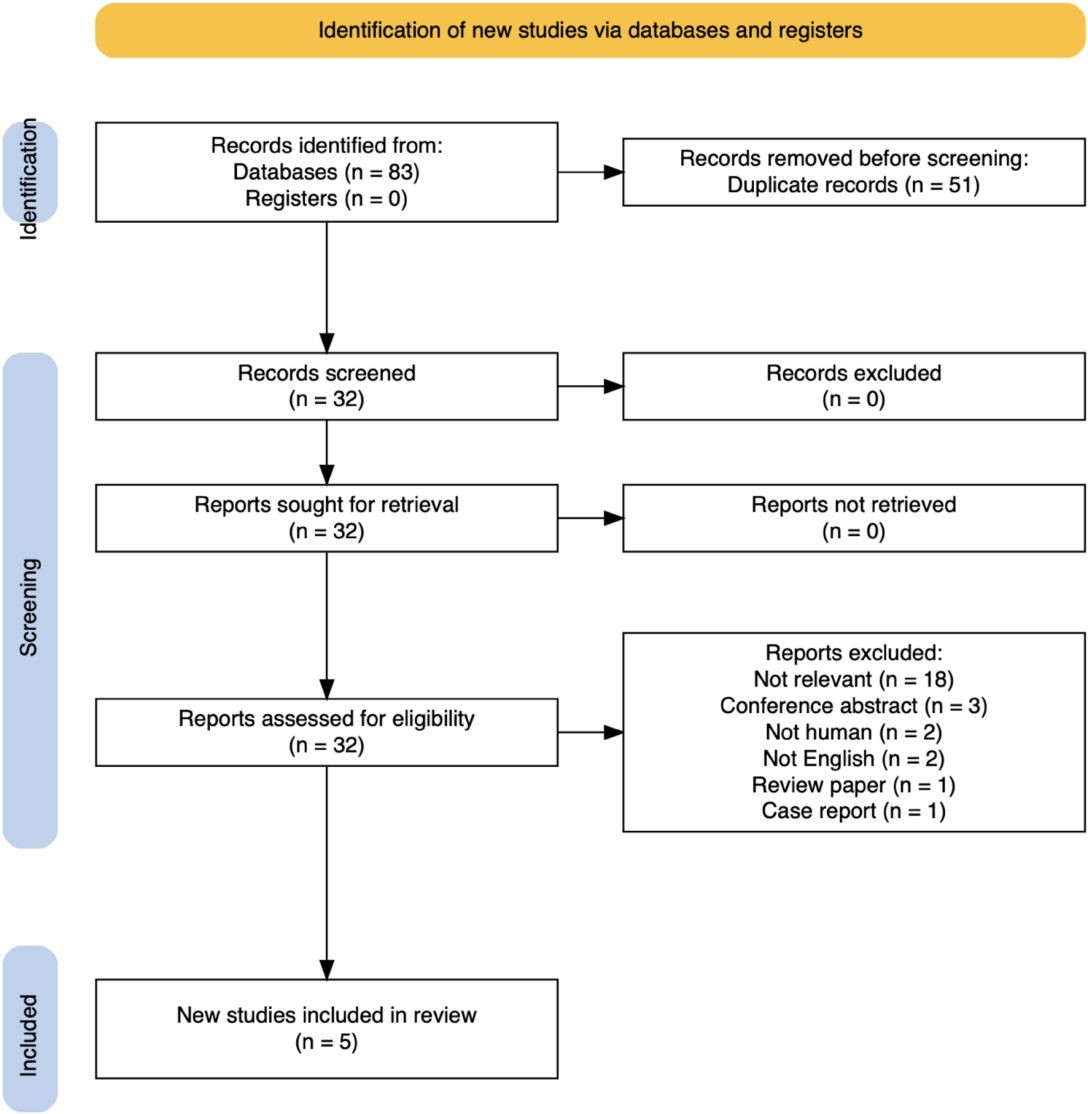
PRISMA chart detailing search strategy.

The inclusion criteria were as follows: [1] adults undergoing abdominal surgery for any indication, [2] the use of RTLS for closure of the abdomen with a comparator group, [3] studies in humans and [4] English language. Studies were excluded if they: [1] described cases in animals or [2] were not specific about the surgical closure technique. Review articles, case reports and editorials were also excluded; however, their reference lists were utilised to gather further eligible studies. This is summarised in a PRISMA chart (Figure 1) [10].

### 2.8 Extraction and Management of Data

Two review authors (RM, SH) independently conducted the search strategy. Two review authors (RM and SCM) screened titles and abstracts of all articles to assess suitability against inclusion criteria and extracted all relevant data from the included articles. Discrepancies in data extraction were clarified by discussion and consensus. If needed, a third independent author (SH) was consulted.

For each included article, the approach to surgical closure was described. Any additional characteristics detailed in the article were included as part of the data collection and presented in this review if there was sufficient reporting; these include but were not limited to age, sex, BMI, comorbidities, outcome data and follow‐up period.

### 2.9 Evaluation of Risk of Bias

An independent assessment of the methodology and risk of bias of our eligible studies was performed by two independent authors (SH and SH) upon criteria outlined by the Cochrane's tool for randomised controlled trials (RCT) [11] and the Risk Of Bias In Non‐randomised Studies—of Interventions (ROBINS‐I) assessment tool [12] for observational studies. Disagreements following such assessment were addressed via discussion between the assessors. Where disagreements persisted, an additional author was involved.

### 2.10 Summary Measures and Synthesis

For dichotomous outcome measures (IH, C‐D III or more, wound dehiscence or wound infection), an odds ratio (OR) was calculated as the estimate of effect size. The OR is the odds of an adverse outcome in the RLTS group compared to that in the no RLTS group. An OR of less than one would favour RTLS. For continuous outcome measures (procedure time) the mean difference (MD) was calculated. When mean values were not available, data on median and interquartile range (IQR) were extracted and subsequently converted to mean and standard deviation (SD) using the well‐practised equation described by Hozo et al. [13].

Review Manager 5.4 software was utilised for analyses which involved use of random‐effects modelling. The results of data synthesis for the evaluated outcomes were presented in a forest plot demonstrating 95% confidence intervals (CIs).

The unit of analysis regarding all evaluated outcomes was an individual patient. Where possible, data regarding dropouts, withdrawals and other missing information were recorded. The final analysis was based on the intention‐to‐treat concept.

We evaluated heterogeneity through the calculation of I^2^ using the Cochran Q test (χ^2^). Heterogeneity was subsequently interpreted whereby 0%–25% was mild, 26%–75% represented moderate heterogeneity and 76%–100% represented considerable heterogeneity. Moreover, funnel plots were constructed to investigate publication bias.

To evaluate the source of heterogeneity, we performed sensitivity analyses. The individual effect of each study on outcomes was evaluated using a leave‐one‐out sensitivity analysis (repeating the outcome synthesis after exclusion of one study at a time).

### 2.11 Ethical Approval

This study is a meta‐analysis of previously published data and did not involve direct patient contact or the collection of individual patient data. As such, formal ethics approval was not required.

## 3 Results

### 3.1 Study Selection

A search across the included databases yielded 83 articles of which 51 were excluded on account of duplication and a further 26 excluded due to being not relevant (*n* = 18), conference abstracts (*n* = 3), in animals (*n* = 2), not English (*n* = 2), review paper (*n* = 1) or a case report (*n* = 1). Ultimately, five studies were included—four randomised controlled trials (RCTs) and one retrospective observational study [14, 15, 16, 17, 18] (Figure 1 and Table 1).

**TABLE 1:** Included studies related data.

Author	Year	Country	Journal	Type of study	Procedure performed
Agarwal	2011	India	*Tropical Doctor*	RCT (single centre)	90 RTLS; 100 PSC
Lozada Hernandez	2022	Mexico	*Hernia*	RCT (single centre)	51 RTLS; 53 PSC
Wenzelburg	2023	Sweden	*Journal of Abdominal Wall Surgery*	Retrospective Observational study (single centre)	47 RTLS; 82 PSC
Wenzelburg	2024	Sweden	*British Journal of Surgery*	RCT (two centre)	80 RTLS; 80 PSC
Lozada Hernandez	2024	Mexico	*Surgical Endoscopy*	RCT (five centre)	125 RTLS; 125 PSC *plus* mesh

Abbreviations: PSC, primary suture closure; RCT, randomised control trial; RTLS, reinforced tension line suture.

The baseline characteristics of included patients are summarised in Table 2 with risk factors for incisional hernia formation and study inclusion and exclusion criteria. Participants were included if high‐risk for incisional hernia for a variety of reasons, including undifferentiated emergency laparotomy, cancer‐related laparotomy, or high‐risk according to the Rotterdam or Modified‐Rotterdam Risk Model [19, 20]. Table 3 details the differences in closure technique between studies, including suture and needle size. The study definition of incisional hernia was described in only one study, and three studies used CT diagnosis in addition to clinical examination (Table 3). Two studies used a small bite technique, with the remaining using large needles and heavy sutures. Four studies described using the Jenkins 4:1 rule for closure.

**TABLE 2:** Baseline characteristics of the included study population.

Author	Year	Sample size, *n* (total)	Sample size, *n* (variable)	Age, mean	Gender distribution, *n*	BMI, mean	COPD, *n*	Smoking status, *n*	Malignant diagnosis, *n*	Previous incision, *n*	ASA > 3, *n*	Emergency procedure, *n*	Follow up, months	Inclusion criteria	Exclusion criteria
Agarwal et al.	2011	190											< 1	Emergency midline laparotomy (benign or malignant)	—
RTLS			90	—	—	—	—	—	—	—	—	90			
PSC			100	—	—	—	—	—	—	—	—	100			
Hernandez et al.	2022	104											36	Emergency or elective midline laparotomy High risk (Rotterdam risk model score > 6^a^)	Pregnant Non‐protocol closure Previous midline laparotomy Relaparotomy during F/U for non‐related cause
RTLS			51	58	29 M, 22F	25.52	11	—	29	—	—	21			
PSC			53	53	26 M, 27F	25.81	9	—	26	—	—	25			
Wenzelburg et al.	2023	129											12	CRS/HIPEC via a midline laparotomy	Non‐protocol closure Existing midline mesh or hernia Deceased or re‐operated within 9 months (any reason) No CT at 12 ± 3 months
RTLS			47	54	24 M, 23F	26.3	3	—	47	13	9	—			
PSC			82	59	38 M, 44F	25.8	5	—	82	25	23	—			
Wenzelburg et al.	2024	160											12	Midline laparotomy for colorectal cancer	Planned cytoreductive surgery/HIPEC Previous midline hernia surgery or current midline hernia ASA PS grade >III Need for fascial reconstruction Peritoneal carcinomatosis No CT scan at 12 ± 3 months Re‐operated within 9 months for any reason
RTLS			80	70	42 M, 38F	26	8	12	80	38	32	—			
PSC			80	67	44 M, 36F	25	7	8	80	45	22	—			
Hernandez et al.	2024	250*												Emergency or elective midline laparotomy High risk (modified Rotterdam risk model score > 4^b^)	Pregnant Non‐protocol closure Previous midline laparotomy Relaparotomy during follow‐up for non‐related cause Life‐expectancy < 12 months
RTLS			125	53.9	65 M, 53F	27.8	6	2	38	—	46	58	1		

Abbreviations: ASA PS, American Society of Anesthesiologists (ASA) physical status classification; CRS/HIPEC, cytoreductive surgery and hyperthermic intraperitoneal chemotherapy; IH, incisional hernia; PSC, primary suture closure; RTLS, reinforced tension line suture.

* 125 patients in control group had onlay mesh.

^a^ Rotterdam Risk Model includes: age, sex, COPD, ascites, jaundice, anaemia, emergency surgery, type of surgery (oesophagus, gastroduodenal, gallbladder/bile duct, small bowel, large bowel, vascular), coughing and wound infection.

^b^ Modified‐Rotterdam Risk model includes: age, sex, COPD, ascites, jaundice, anaemia (Hb < 12 g/dL), emergency surgery and type of surgery (oesophagus, gastroduodenal, gallbladder/bile duct, small bowel, large bowel, vascular).

**TABLE 3:** Summary of closure techniques and incisional hernia definition from included studies (references in table) with modifications for brevity and clarity. Original descriptions can be found in the cited sources.

Author (year)	Closure technique			Diagnosis and definition of IH
		Control group: PSC closure technique	Intervention group technique: RTLS closure technique	
Agarwal et al. [14]	Suture, needle	Loop PDS on a 65‐mm 1/2 needle *(0 or 1)*	PDS on 65‐mm 1/2 needle *(0 or 1)*	*(Assessed for acute abdominal wall dehiscence)*
	Technique description	A simple continuous mass closure	Longitudinal continuous suture parallel to the linea alba on both sides, after clearing 2 cm of fat. A new continuous suture placed ensuring it is lateral to the longitudinal suture, tied and knotted. The longitudinal suture is made taut and tied	
Lozada‐Hernández et al. [15]	Suture, needle	1 PDS Plus II monofilament, 48 mm needle (Ethicon).	2 PDS Plus II monofilament, 48 mm needle (Ethicon)	Clinical diagnosis (definition not described), CT if diagnosis uncertain
	Technique description	Jenkins 4:1, continuous suture (10 mm from fascial edges, 10 mm apart), knot‐tying separately at the midpoint. The rest of the wound was closed in a conventional manner and no drains were left in the wound	Longitudinal continuous suture parallel to the linea alba on both sides, at 10 mm intervals, 5–8 mm from the aponeurosis edge. The ends of the two sutures were tied at the angles. Wound then closed as per PSC closure technique, ensuring the stitch included and anchored the two longitudinal strands of reinforcement	
Wenzelburg et al. [17]	Suture, needle	2–0 PDS suture	2–0 PP suture	EHS definition ‘*any abdominal wall gap with or without a bulge in the area of a post‐operative scar, perceptible or palpable by clinical examination or imaging*’. CT assessed by three independent examiners (two surgeons, one radiologist). In case of discrepancy discussion undertaken and consensus decision
	Technique description	Jenkins 4:1, continuous suture according to the description by Millbourn et al.	RTL‐suture of PP according to Hollinsky et al. The continuous 4:1 closing suture was placed just outside and including the RTL‐suture in every stitch. The RTL suture was then tied	
Wenzelburg et al. [18]	Suture, needle	2/0 PDS Plus suture on a CT‐2 needle (PDSPlus; Ethicon).	2/0 PP on a CT‐2 needle (Prolene; Ethicon)	Clinical or CT diagnosis. CT assessed by three independent examiners (two surgeons, one radiologist). In case of discrepancy discussion undertaken and consensus decision. IH definition not documented
	Technique description	Jenkins 4:1, small‐bite technique (5–8 mm from the fascial edges, 5 mm apart), only including the fascia. Skin closure with a running intracutaneous 4/0 PDS (PDSPlus)	RTL‐suture of PP along both sides of the incision within the condensed linea alba as described by Hollinsky et al. Fascia dissected free from subcutaneous fat 10 mm outside the incision in all directions. The incision was closed using the 4:1 small‐bite technique according to Millbourn et al.	
Lozada Hernández et al. [16]	Suture, needle	1 PDS Plus II monofilament, 48 mm needle (90 cm; Ethicon)	2 PDS Plus II monofilament, 48 mm needle (90 cm; Ethicon)	*(Assessed for acute abdominal wall dehiscence in 1st 30‐days—clinically or by ultrasound if uncertain)*
	Technique description	*ONLAY MESH CLOSURE: Simple continuous suture from either end, advancing 10 mm along and 10 mm from the aponeurosis edge to the wound, following the Jenkins 4:1 rule. Sutures were knotted separately (self‐locking/Aberdeen knot). As per Lima et al., an anterior plane approximately 8 cm was created between the anterior rectus fascia and the subcutis. An onlay polypropylene mesh was placed on the anterior rectus fascia with an overlap of 3 cm, fixed to the underlying fascia with multiple running sutures (polyglactin 2/0). Suction drainage was used above the mesh, and the drain was removed when the flow rate was < 50 mL/day. Subcutaneous tissue and skin were closed with sutures preferred by the surgeon*	As described by Lozada‐Hernández et al. [15], longitudinal continuous suture parallel to the linea alba on both sides, at 10 mm intervals, 5–8 mm from the aponeurosis edge. Subcutaneous tissue and skin were closed with sutures preferred by the surgeon. No drains were left in the wound	

Abbreviations: IH, incisional hernia; PDS, polydioxanone suture; PP, polypropylene; PSC, primary suture closure; RCT, randomised control trial; RTLS, reinforced tension line suture.

### 3.2 Methodological Appraisal

**FIGURE 2:**
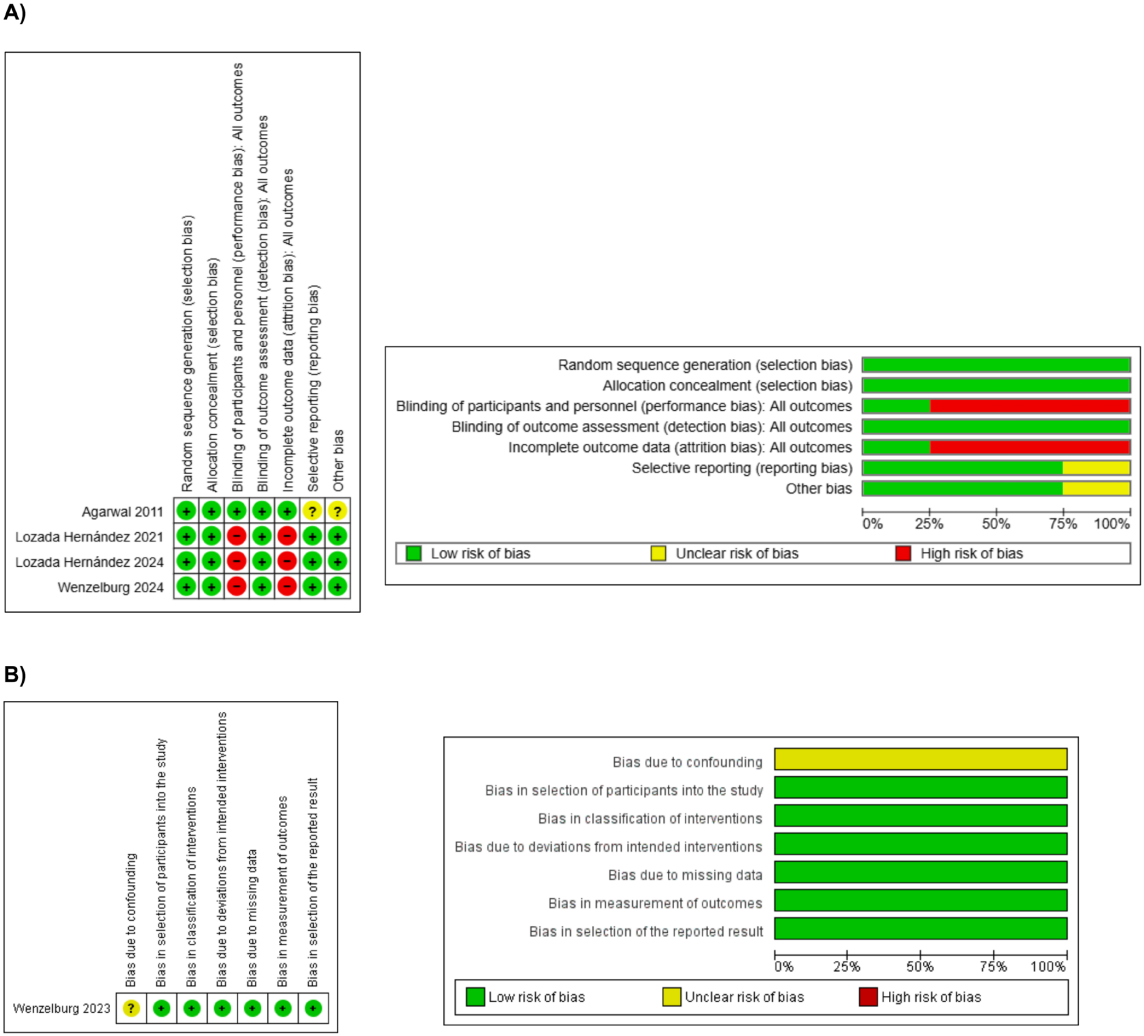
Risk of bias assessment (A) randomised controlled trials (B) observational studies.

The risk of bias assessment of the eligible four RCTs is outlined by Figure 2A. All four RCTs had a low risk of selection bias and detection bias. The risk of performance bias was low in one study and high in three studies. The risk of attrition bias was low in one study and high in three studies. The risk of reporting bias was low in three studies and unclear in one study. The risk of other types of bias was low in three studies and unclear in one study.

Figure 2B outlines the outcome of the risk of bias assessment of the included observational study. The study was associated with an unclear risk of bias due to confounding whilst the risk of other types of bias was low.

### 3.3 Outcome Synthesis

**FIGURE 3:**
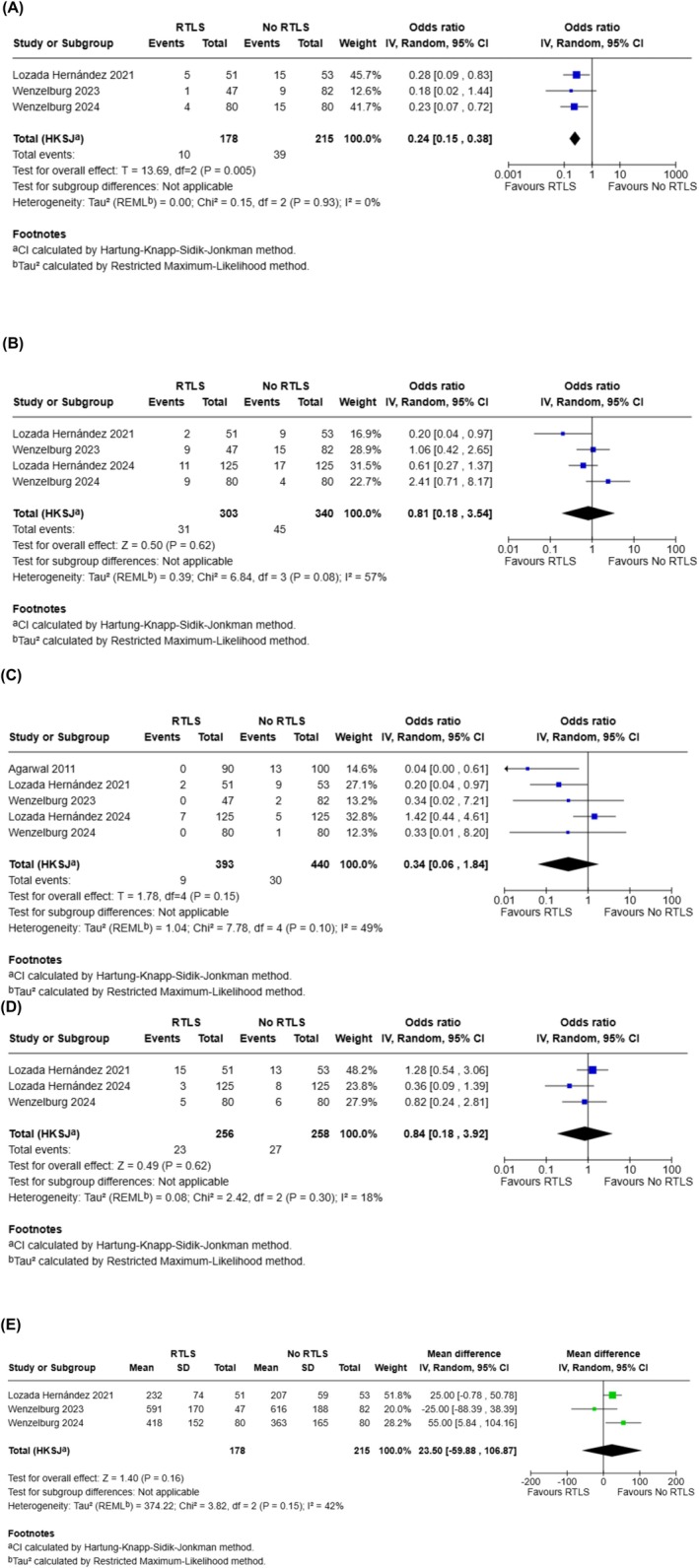
Forest plots of comparison for: (A) Incisional Hernia; (B) post‐operative complications (Clavien‐Dindo≥III); (C) wound dehiscence; (D) wound infection; and (E) procedure time. The solid squares denote the odds ratio (ORs) or mean difference (MD). The horizontal lines represent the 95% confidence intervals (CIs), and the diamond denotes the pooled effect size. M–H, Mantel–Haenszel test.

Outcomes are summarised in Figure 3.

*Post‐operative incisional hernia (IH)*: Three studies (393 patients) were included in the analysis of post‐operative IH. The rate of post‐operative IH in the RTLS group was 5.6% and it was 18.1% in the no RTLS group. Use of RTLS was associated with a significantly lower rate of post‐operative IH (OR 0.24; 95% CI: 0.15–0.38, *p* = 0.005). Low heterogeneity was detected amongst the included studies (I^2^:0%, *p* = 0.93).

*Clavien‐Dindo complications (C‐D) ≥ III*: Four studies (643 patients) were included in the analysis of C‐D ≥ III. The rate of C‐D ≥ III in the RTLS and no RTLS groups was 10.2% and 13.2%, respectively. No significant difference was found in C‐D ≥ III between the two groups (OR 0.81; 95% CI: 0.18–3.54, *p* = 0.62). Moderate between‐study heterogeneity was detected (I^2^:57%, *p* = 0.08).

*Wound dehiscence*: Five studies (833 patients) were included in the analysis of wound dehiscence. The rate of wound dehiscence in the RTLS and no RTLS groups was 2.3% and 6.9%, respectively. No significant difference was found in wound dehiscence between the two groups (OR 0.34; 95% CI: 0.06–1.84, *p* = 0.62). Heterogeneity amongst the included studies was moderate (I^2^:49%, *p* = 0.10).

*Wound infection*: Three studies (514 patients) were included in the analysis of wound infection. The rate of wound infection in the RTLS and no RTLS groups was 9.0% and 10.4%, respectively. No significant difference was found in wound infection between the two groups (OR 0.84; 95% CI: 0.18–3.92, *p* = 0.62). Heterogeneity amongst the included studies was low (I^2^:8%, *p* = 0.30).

*Procedure time*: Three studies (393 patients) were included in the analysis of procedure time. The mean procedure time in the RTLS and no RTLS groups was 413.6 ± 146.5 and 329.3 ± 203.4 min, respectively. No significant difference was found in procedure time between the two groups (MD 23.50; 95% CI: −59.88–106.87 *p* = 0.16). Moderate heterogeneity was detected amongst the included studies (I^2^: 42%, *p* = 0.15).

### 3.4 Sensitivity Analysis

The direction of pooled effect size remained unchanged when the risk ratio (RR) or risk difference (RD) was calculated. During leave‐one‐out sensitivity analysis, removal of the Lozada Hernández 2024 study in the analysis of wound dehiscence made the results significant in favour of RTLS (OR 0.17; 95% CI: 0.04–0.68, *p* = 0.03) and reduced degree of heterogeneity from 49% to 0%.

## 4 Discussion

In view of growing evidence in favour of the effectiveness of the RTLS technique for closure of high‐risk midline laparotomy, a comprehensive systematic review and meta‐analysis of the best available evidence was conducted and five comparative studies were included, reporting a total of 708 patients of whom 393 had their laparotomy wound closed using RTLS and the remaining 315 patients had standard closure. Evaluation and analysis of the data suggested that RTLS significantly reduced the risk of IH compared to the standard technique. However, no significant differences were found in C‐D ≥ III complications, wound dehiscence, wound infection or procedure time between the two groups. Between‐study heterogeneity was low or moderate in all the outcome syntheses which suggests the robustness of our findings.

Abdominal wall closure techniques have evolved over time with a greater focus on targeted closure to prevent IH, especially in high‐risk patients. Though many techniques have been reported across the literature, most focus on modified suture repairs or the introduction of prophylactic mesh. Alternative closure strategies have been investigated, such as the HART trial investigating the role of the Hughes closure technique (mass closure large‐bite primary suture closure [PSC] with added interrupted nylon double‐far‐near‐near‐far sutures) in the prevention of post‐operative IH against standard PSC, which ultimately demonstrated no significant effect with a 1‐year IH rate of 14% Hughes closure versus 17% standard PSC, increasing to 28% and 31% at 2 years [6]. The STITCH trial compared small versus larger bite midline abdominal closure and demonstrated a significant reduction in the incidence of post‐operative IH from 21% to 13% after 1‐year follow‐up in the small‐bite group (35 out of 268 patients, *p* = 0.0220) [7]. Despite these promising results, the STITCH trial has not been adopted into clinical practise. Barriers to its implementation are likely to include increased operative time needed to facilitate smaller bites or an aversion to change in clinical practise. Additionally, the difference in incidence of post‐operative IH (though statistically significant) may seem too marginal to motivate a change in practise or convince of efficacy, and longer‐term data, or health‐economic benefit and patient‐reported outcome measures may prove a more meaningful outcome to clinicians to change practise. A potential avenue for further investigation is the combination of small bite sutures as well as RTLS with the aim to establish synergistic tension redistribution and thus reducing post‐operative IH more effectively than either technique alone. The beneficial impact of the RTLS technique may be further strengthened by the consistent use of the small‐bite technique in combination with the RTLS, as only two studies in this meta‐analysis used the small‐bite technique; yet still, a significant difference was demonstrated.

RTLS may offer a more targeted approach in the prevention of post‐operative IH in high‐risk patients; therefore, defining this cohort is key in its application. Pereira‐Rodriquez et al. classified high‐risk groups as those which were related to the patient (such as age or obesity), the situation (such as in emergency surgery, redo procedures or those requiring an ostomy), or the pathology (such as in resection of intra‐abdominal malignancy or liver transplantation) [2]. Additionally, application of score systems such as the HERNIAscore or the PENN hernia risk calculator may also be useful in defining these high‐risk patients for whom an RTLS supported closure would lower their cumulative risk of post‐operative IH [21, 22]. Whilst high‐risk cohorts are generally well described across the literature, certain subgroups have been underrepresented or inadequately reported, and these include patients with chronic immunosuppression, connective tissue disorders, cirrhosis or frailty syndromes. As such, the generalisability of the RTLS approach to high‐risk patients is not confident. Beyond the application to high‐risk patients, there are no large‐scale direct comparisons of RTLS against other prophylactic strategies, such as prophylactic mesh techniques, which further limits the ability to determine if RTLS provides a distinct advantage or if it should be incorporated into a multimodal closure approach [6]. Whilst RTLS is particularly promising, high‐quality randomised trials with rigorous risk stratification are essential in refining patient selection criteria and optimising IH prevention strategies.

Although our findings did not demonstrate any significant difference in wound‐related complications between RTLS and other techniques, a 4.5% increased risk of wound dehiscence associated with standard closure may be clinically important, albeit statistically insignificant. Furthermore, during our sensitivity analysis, removal of the source of heterogeneity in the analysis of wound dehiscence made the results significant in favour of RTLS. Future RCTs may provide stronger evidence in favour of RTLS in this context.

It is important to consider several limitations that are inherent to this paper. Although the risk of selection and detection bias of the studies was low, two RCTs had a high risk of performance bias and unclear risk of reporting bias in one study. Additionally, whilst the differences in IH rates were significant between RTLS and no RTLS, it is important to consider the underlying factors that may have contributed to this difference including patient factors, surgical technique and post‐operative course which were not consistently reported across the included articles. It is important to note also that the generalisability of the results is limited in the setting of small sample sizes and limited follow up periods. Notably, Agarwal et al., and Hernandez et al. both had short follow up periods reporting abdominal dehiscence rather than post‐operative IH, which limited the patient cohort for IH assessment [14, 16]. Further, Agarwal et al. lacked detailed descriptions of patient characteristics and those which would be otherwise used to determine high‐risk groups, making it difficult to assess the applicability of RTLS in these cohorts [14]. Most importantly for assessment of a surgical technique, studies did not consistently use the small‐bite closure technique, introducing additional variability into the assessment. Only two studies reported small‐bite closure; therefore, the effect of RTLS could be further strengthened if future assessment uses small‐bite closure as the standard closure and RTLS technique. Ultimately, these limitations highlight the need for rigorous, longitudinal studies to further objectively characterise the efficacy of RTLS in the long‐term prevention of post‐operative IH.

## 5 Conclusion

Incisional hernia remains a common post‐operative complication of abdominal surgery. RTLS is an emerging closure technique that aims to decrease the mechanical stress along a tension line when compared to no RTLS. This systematic review demonstrates that RTLS may offer an advantage in reducing the risk of developing incisional hernias in comparison to no RTLS, whilst also retaining a similar safety profile. Larger, multi‐centre studies are warranted to validate these findings and define the high‐risk patient groups that benefit from the technique and assess the long‐term efficacy, cost effectiveness and impact on patient quality of life of RTLS as a standard technique for abdominal wall closure.

## Disclosure

The authors have nothing to report.

## Appendix A

**Table:**

Search no	Search strategy^a^
#1	MeSH descriptor: (laparotomy) explode all trees
#2	Laparotomy: TI, AB, KW
#3	MeSH descriptor: (abdominal surgery) explode all trees
#4	Abdominal surgery: TI, AB, KW
#5	#1 OR #2 OR #3 OR #4
#6	MeSH descriptor: (tension‐line sutures versus) explode all trees
#7	Tension‐line sutures versus: TI, AB, KW
#8	MeSH descriptor: (closure) explode all trees
#9	Closure: TI, AB, KW
#10	#6 OR #7 OR #8 OR #9
#11	#5 AND #10

^a^ This search strategy was adopted for following databases: PubMed, MEDLINE, and Web of Science.
